# On the Causes of Irritability and Excitability

**Published:** 1803-09-01

**Authors:** M. De La Melhere


### *On the* Causes *of* Irritability *and* Excitability; *by* M. De La Melhere.

[ Continued from p. 25. — 29. ]

OTHER physiologists have imagined they found in the structure of nerves the cause of irritability and excitability. All the mechanical theories founded on the distention by fluids are abandoned, and we may add, that the fibrine, which cannot be said to possess either nerve, nervous fluid, or muscles, possesses excitability. We must, therefore, look for some other cause of contraction; and the author supposes, that it is the same which produces the contraction of a piece of leather, on which acid or fire acts; he thinks that it is a real crispation caused by the electric fluid, and which acts on it in the same way that the pile of Volta does when it oxydates metals; but he asks, How does the electric or Galvanic spark produce this crispation of the fibre or the oxydation of metals? We must, says he, consider the animal fibre as composed of a glutinous matter, which becomes thinner and shorter on being dried; and which swells and becomes longer on being moistened. The electric or Galvanic spark produces sufficient heat to crisp and contract the fibre, since it can oxydate metal. Rumford has proved, that fluids were bad conductors of heat; from which he concludes that a strong heat may exist even where it is not procured; he thinks, for instance, that a ray of light striking in the middle of water, a body which is a conductor of heat, such as muriate of silver, will produce on each of its particles heat sufficient to disengage its oxygen. He has also found, that when the rays of the sun fall on a solution of gold, that the metal is revivified; here we must suppose the production of heat. These facts, the author thinks, authorize us to conclude, that in Galvanism, the spark which penetrates the body of the animal, animal, acts only on the solid parts, and that the heat is strong enough to crisp and produce contraction of the fibre. We have further seen that the nerves, muscles, and viscera of animals, form a Galvanic pile, in which continual discharges are made of electric fluid, from nerves to muscles, to the viscera, and from muscles to nerves; these discharges are accompanied by heat, as in the electric discharge. The heat being surrounded by fluids, which are non-conductors, is determined to the solid parts, i. e. the fibre, on which it produces a momentaneous crispation, as it oxydates metals in the pile of Volta, although it be placed in water, or surrounded with cloths. In this way only, M. De la Melhere, thinks can be explained the contraction of the fibrine of the blood, when it is submitted to the positive and negative poles of the pile, for it is found to crisp as if exposed to heat.*

The nervous fluid, and the structure of nerves, also produce a peculiar action on the phenomena just now mentioned. Let us suppose with Reil, the nerve made up of nerves, making infractuosities, and in which is lodged the medullary substance; let us also suppose this substance to contain a quantity of Galvanic fluid; this medullary substance, and the fluid which it contains, will furnish a considerable quantity of electric fluid to act on the nerves. Their structure will increase this contraction. It is conceived in this hypothesis, that the nerves are absolutely necessary to the motions and sensibility of animals, since it is they which carry to the muscles and viscera this substance impregnated with Galvanic fluid, without which the animal would experience a momentaneous excitement only, as we find to be the case in the detached parts of animals. The circulation becomes necessary by keeping up vital heat, which is necessary to Galvanism.

### *Of the Cause of Motion and Sensation in Animals.*

From what has been just said we may suppose, that in the natural state of an animal there is a state of equilibrium.

---

* This I think a very probable explanation of this, otherwise very singular, phenomenon. Having been present at the experiments, from which the irritablility of the fibrine was inferred, I can affirm that the contractions or motions which were perceivable, resembled extremely those which heat does produce on similar substances; in giving testimony, however, to this fact, it is not intended either to admit the analogy between the phenomena produced by electricity on the fibrine and muscular contraction, nor to assent in any manner to his explanation of that property in animal matter.

brium between the Galvanism of the nerves and that of the muscles and other parts, as in the Leyden phial charged. The following is the manner in which the author thinks of the mechanism of motion and sensation. A sensation is produced only when a substance strikes the surface of the body, as in the sense of touch. The skin of the animal being compressed, as it contains many nerves, dispersed on a kind of aponeurotic substance, the equilibrium is destroyed by this contact, exactly as takes place when the torpedo is touched; the consequence is a Galvanic discharge. Under common circumstances the discharge is too slight to produce a commotion similar to what takes place in the torpedo, yet it is sufficiently strong to produce a commotion in the nerve to its very origin in the brain, where we suppose the sensitive principle exists. This impression continues, as in the Galvanic pile, as long as the body, which touched, remains on the skin. As often as this discharge affects the sensitive principle, a sensation is produced. This electric discharge bringing into play the excitability of the nerves, crisps and contracts them as well as the muscles to which they are distributed, and the animal is moved by the same cause which moves frogs and other animals submitted to Galvanic experiment.

The phenomena which the discoveries on Galvanism have developed, and the interest which the subject, from its novelty excites, are the motives which have induced us to deviate from our usual plan of confining our communications to practical subjects, or at least such as have a less visionary foundation. It may not be thought unimportant to know the speculations of a man who ranks extremely high among the philosophic men of France.

